# Feature Optimization for Long-Range Visual Homing in Changing Environments

**DOI:** 10.3390/s140203342

**Published:** 2014-02-19

**Authors:** Qidan Zhu, Xue Liu, Chengtao Cai

**Affiliations:** College of Automation, Harbin Engineering University, Harbin 150001, China; E-Mails: zhuqidan@hrbeu.edu.cn (Q.Z.); caichengtao@hrbeu.edu.cn (C.C.)

**Keywords:** long-range visual homing, uniform distribution, feature selection, feature updating, changing environments

## Abstract

This paper introduces a feature optimization method for robot long-range feature-based visual homing in changing environments. To cope with the changing environmental appearance, the optimization procedure is introduced to distinguish the most relevant features for feature-based visual homing, including the spatial distribution, selection and updating. In the previous research on feature-based visual homing, less effort has been spent on the way to improve the feature distribution to get uniformly distributed features, which are closely related to homing performance. This paper presents a modified feature extraction algorithm to decrease the influence of anisotropic feature distribution. In addition, the feature selection and updating mechanisms, which have hardly drawn any attention in the domain of feature-based visual homing, are crucial in improving homing accuracy and in maintaining the representation of changing environments. To verify the feasibility of the proposal, several comprehensive evaluations are conducted. The results indicate that the feature optimization method can find optimal feature sets for feature-based visual homing, and adapt the appearance representation to the changing environments as well.

## 1. Introduction

A usual dichotomy partitions robot visual navigation methods into quantitative and qualitative methods [1]. The estimation of metric position information is not needed in a qualitative method, and the environments are usually represented by a route. It is crucial for robots to move autonomously from their current position to the home position along the route. Numerous methods for route guidance have been proposed, including node-based [2–6] ones and those that do not use nodes [7,8]. Navigation models that do not separate routes into sequences of waypoints have been proposed in [7,8], following the widely neglected branch of models based on recognition-triggered response. The navigation with nodes has been modeled by local visual homing strategies [2–5]. Inspired by the simple principle of biological navigation, local visual homing has the ability to return to a previously visited location, and has drawn attention in robotics study as computationally cheap building blocks in a minimalistic appearance-based navigation framework.

In this paper, we present that long-range homing along a route can be achieved by implementing local visual homing strategy to sequentially visit the topological nodes. In the feature-based local visual homing in unprepared environments, the extracted visual features are ideal alternatives to artificial landmarks. However, a big challenge during long-range homing in changing environments is the management of rich feature sources [9,10]. In order to avoid irrelevant and redundant features, task-oriented selection schemes are needed, which contain additional constraints in evaluating feature usefulness for the tasks. Furthermore, in order to obtain optimal features during long-range navigation, it is necessary to maintain an up to date representation of surrounding environments. Propelled by above requirements, the main work of this paper focuses on the feature optimization orienting towards long-range feature-based visual homing in changing environments.

Several feature-based visual homing strategies have been presented in the literature, such as average displacement vector [11], average landmark vector (ALV) [12] and the included angle difference method [2]. However, they all need or imply an equal-distance assumption that all landmarks are distributed at the same distance from the snapshot location. Since the assumption is always violated in unstructured environments, the true home vector will deviate from the estimated direction [2,11,13]. Uniformly distributed features do improve the feature-based homing accuracy, especially for the ALV, which has drawn significant attention for simplicity [13]. There are adequate visual features extracted from unstructured environments to identify a goal to home, yet no attention has been given to modify the feature distribution for feature-based visual homing. A few of the widely employed feature extraction algorithms have tried to get uniformly distributed features [14–16], however, none meets the requirement on feature distribution for feature-based visual homing.

The feature selection is essential to object classification [17], localization [18], robot navigation [19,20], *etc.* With respect to the features used in visual homing, there are several criteria for the selection process [21,22], in which the demanding task is the explicit quantitative characterization of feature properties in view of their relative importance. In the previous literature, most approaches to the characterization of feature quality focus on recognition and classification tasks, but few of them are ideally suited to feature-based visual homing. Furthermore, most previous research on visual homing was carried out under the assumption that the environments are static. The significance of rating and updating mechanisms, which are crucial to continuously evaluating the relative importance of features and discarding useless ones, is always ignored.

Motivated by the aforementioned thought, our work concerns the optimization of feature distribution, selection and updating. In particular, we focus on acquiring uniformly distributed features to fulfill the equal-distance assumption. The features are graded by the quantitatively characterized selection criteria of visual homing. When the agent retraces the environments, the importance of features is re-evaluated to update the appearance representation. In this paper, the ALV strategy is adopted as building blocks of the route for simplicity. Besides, the features are extracted by SURF algorithm [23], because of its high accuracy and less computing time. In order to improve the performance of long-range feature-based visual homing in changing environments, the work presented in this paper concentrates on maximizing the advantage of the ALV method by modifying the distribution of high quality SURF features.

The remainder of the paper is organized as follows: Section 2 outlines the extraction algorithm of well-distributed SURF features. Section 3 presents the feature selection and updating mechanisms. Section 4 shows the framework of feature optimization. Experiments in Section 5 demonstrate the performance. Section 6 draws conclusions and points out future work directions.

## 2. Uniformly Distributed Features

Due to their good invariance, local features have been introduced to substitute for artificial landmarks when the agent is situated in unknown environments. The Speeded Up Robust Features (SURF) [23] and Scale-Invariant Feature Transform (SIFT) [24] are widely used, however, neither controls the feature distribution. Several modified versions have focused on improving spatial distribution [14–16], but none can ideally meet the need of feature-based visual homing. In addition, little work has been done to improve the distribution of SURF features. Considering that SURF burden is computationally low, we aim at improving the spatial distribution.

**Figure 1.**
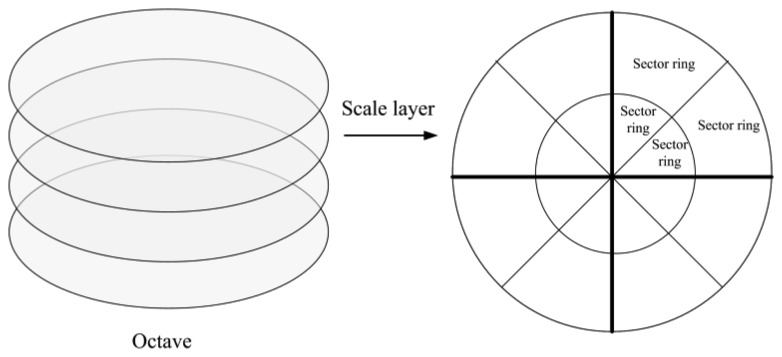
The octave and division of the scale layer. The left side shows the octave consisting of scale layers. The right side shows the scale layer consisting of 16 sector rings.

Considering that the SURF features are extracted in scale space and the image sizes are identical, extracting features over the scale space in a uniform way becomes very important. The SURF scale space consists of several octaves and scale layers, as shown in the left side of Figure 1. In consideration of using panoramic images, each scale layer can be divided into regular sector rings along the radial and circumferential directions (right side of Figure 1). The features should be uniformly distributed in each sector ring. Therefore, the feature distribution problem in the image can be seen as a problem in scale layers. Since the scale space is constructed by up-scaling the box filter size, the number of features decreases as the filter size increases because of the smoothing characteristics. Besides, the number of sector rings decreases progressively among octaves of the scale space.

Supposing that the number of key-points extracted by the standard SURF algorithm in the scale layer (*s*) of the octave (*o*) is *N_os_*, the number of sector rings is *n_os_*, and the key-point number in the *i*th sector ring is $M_{\text{os}}^{i}$. The feature distribution in the scale layer will be relatively uniform if the key-point number in each sector ring is the same. We can define $N_{\text{os}}^{i}$ in Equation (1), which denotes the target number of key-points when each sector ring has the same key-point number. For the *i*th sector ring, all the key-points in this sector ring will be reserved when $M_{\text{os}}^{i}$ is not greater than $N_{\text{os}}^{i}$. On the contrary, if $M_{\text{os}}^{i}$ is greater than $N_{\text{os}}^{i}$, the excess key-points will be discarded by their quality which is measured according to strength value and spatial dispersion. The strength value *V_str_* will be shown in Equation (16) and specifically explained in Section 3.1. With regard to spatial dispersion, it is computed as follows. As shown in Equation (2), *E_j_* is the entropy of the square region used to construct the descriptor of the *j*th key-point, and *q_l_* is the probability of the *l*th gray level value over all the grey levels within the square region:

(1) $$\begin{array}{cccc} N_{os}^{i}=\frac{N_{os}}{n_{os}}, & o=1,2,\ldots ,O; & s=1,2,\ldots ,S; & i=1,2,\ldots ,n_{os} \end{array}$$

(2) $$E_{j}=-\underset{l}{\text{∑}}q_{l}\log_{2}\;q_{l}$$

Supposing (*x_j_*, *y_j_*) is the position of the *j*th key-point in the *i*th sector ring. The center (*x̄, ȳ*) of all the key-points in the *i*th sector ring can be computed by Equation (3):

(3) $$\left(\bar{x},\bar{y})=(\frac{\overset{M_{os}^{i}}{\underset{j=1}{\text{∑}}}E_{j}x_{j}}{\overset{M_{os}^{i}}{\underset{j=1}{\text{∑}}}E_{j}},\frac{\overset{M_{os}^{i}}{\underset{j=1}{\text{∑}}}E_{j}y_{j}}{\overset{M_{os}^{i}}{\underset{j=1}{\text{∑}}}E_{j}}\right)$$

where *E_j_* is taken as the weight of the *j*th key-point. Supposing *dist_j_* in Equation (4) is the Euclidean distance between the *j*th key-point and the center, and *Dist* in Equation (5) is the divergence factor of the *i*th sector ring. Then the quality of the *j*th key-point in the *i*th sector ring can be measured by its strength value and spatial dispersion, as given in Equation (6) where *W_D_* is weight factor:

(4) $${\text{dist}}_{j}=\sqrt{{\left(x_{j}-\bar{x}\right)}^{2}+{\left(y_{j}-\bar{y}\right)}^{2}}$$

(5) $$\text{Dist}=\sqrt{\frac{\overset{M_{os}^{i}}{\underset{j=1}{\text{∑}}}{\left(x_{j}-\bar{x}\right)}^{2}+\overset{M_{os}^{i}}{\underset{j=1}{\text{∑}}}{\left(y_{j}-\bar{y}\right)}^{2}}{M_{os}^{i}}}$$

(6) $$U_{j}=W_{D}\frac{{\text{dist}}_{j}}{\text{Dist}}+(1-W_{D})\frac{V_{\text{str}}^{j}}{\frac{1}{M_{os}^{i}}\overset{M_{os}^{i}}{\underset{k=1}{\text{∑}}}V_{\text{str}}^{k}}$$

In order to acquire uniformly distributed key-points, the points with lower quality are rejected to make sure that the key-point number is less than $N_{\text{os}}^{i}$ in each sector ring. A pseudo-code description of the extraction of uniformly distributed features is given in Algorithm 1.


**Algorithm 1** Algorithm of uniformly distributed feature extraction.
 1:
$M_{\text{os}}^{\text{i}}$ is set as the original key-point number of the *i*th sector ring in scale layer (*s*) of octave (*o*) 2:
$N_{\text{os}}^{\text{i}}$ is set as the key-point number of the *i*th sector ring in scale layer (*s*) of octave (*o*) when uniform distribution 3:**for** (each scale layer of each octave) **do** 4: Calculate 
$N_{\text{os}}^{\text{i}}$ according to Equation (1) 5: **for** (each sector ring) **do** 6:  Check whether 
$M_{\text{os}}^{\text{i}}$ is greater than 
$N_{\text{os}}^{\text{i}}$. 7:  **if** TRUE **then** 8:   Calculate the key-point quality according to Equation (6). 9:   Classify the key-points by their quality in descending order. 10:   Discard the last (
$M_{\text{os}}^{\text{i}}$−
$N_{\text{os}}^{\text{i}}$) key-points. 11:  **else** 12:   Reserve all the key-points. 13:  **end if** 14: **end for** 15:**end for**


## 3. Proposed Approaches to Feature Selection and Updating

The feature strength is crucial in feature-based visual homing in unstructured environments where adequate visual features can be extracted. With respect to the features used in the homing process, there should be several criteria of feature selection to evaluate their strength, in addition to the feature distribution. Besides, the fulfillment of long-range navigation in changing environments critically depends on the feature updating.

### 3.1. Feature Selection

Besides the criterion mentioned above, there are three more criteria identified for selection of features [21,22]: (1) *relevance*: the relevance of a feature is defined as its importance in making navigational decisions; (2) *distinctiveness*: features should be clearly distinguishable from their surroundings, which means the feature configurations should be unique; (3) *reliability*: taking the invariance of feature descriptor into account, features should cope with normal image deformations, which requires high robustness. What really counts is that the features should be widely visible, and consistently observed when the robot passes the same position every time.

The next step is to get the quantitative characterization of the criteria, in order to assign individual strength value to each feature. From the *relevance* aspect, individual feature evaluation is based on their relevance to the task and relationships with other features. Relevance and relationships are generally characterized in terms of correlation and mutual information. The distribution quality, discussed in Section 2, is significant for visual homing and has been taken into account during feature selection process, thus every selected feature is surely relevant to the homing task. The mutual information has been applied for navigation tasks [6,20,25,26], however, it is used in a different way in this paper. The mutual information can be used to measure the quantity of information shared by two random variables. Therefore, it can be used to evaluate the relationships among features. The more information it measures, the more relevant the feature relationships are. The mutual information is defined as follows:

(7) MI(X,Y)=H(X)−H(X|Y)

where the entropy *H*(*X*) measures the variability of variable *X. H*(*X*∣*Y*)is the conditional entropy, giving the expected information in *X* if a measurement is taken.

Supposing *C* denotes the group of all the *T* features in one sector ring. The mutual information *MI*(*C*,*F*) between group *C* and feature *F* in this group can be measured by Equation (7). Due to the lack of previous knowledge, it is supposed that *p*(*C*) has a uniform prior. Therefore, all the *T* features in group *C* have the same probability and the entropy of group *C* can be computed by Equation (8). In addition, the conditional entropy is given by Equation (9):

(8) $$H(C)=\log_{2}T$$

(9) $$H(C|F)=-p(C|F)\times \log_{2}(p(C|F))$$

where *p*(*C*∣*F*) can be computed by the Bayes rule:

(10) $$p(C|F)=\frac{p(F|C)p(C)}{p(F)}$$

Here, *p*(*C*∣*F*) is estimated by applying a kernel density estimator [27]:

(11) $$p(F|C)=\frac{1}{T}\overset{T}{\underset{i=1}{\text{∑}}}K(d(F,F_{i}))$$

In Equation (11)*K*(·); is the Gaussian Kernel function with standard deviation, and *d*(·)); denotes the Euclidean distance. An efficient and accurate nearest-neighbor approximation of this estimator is done as follows [27]:

(12) $$p(F|C)=\underset{T}{\max}K(\text{dist}(F,F_{i}))$$

During the navigation, the images captured at current positions are continuously matched with the image stored at the target. Supposing *M* is the number of times the target image has been matched, and *m* is the number of times that the feature *F* extracted from the target image has been matched. The mutual information of feature *F* can be computed according to Equation (13):

(13) $$MI=\frac{MI(C,F)+\overset{m}{\underset{j=1}{\text{∑}}}MI(C_{j},F_{j})\times e^{-d_{j}}}{m+1}$$

where *F_j_* is the matching feature of *F*, *d_j_* denotes the matching distance between *F* and *F_j_*, and the structural principle of *C_j_* is the same as *C*.

A *distinctiveness* selection can be on the basis that the more informative the feature is, the greater its distinctive power reduces. The feature distinctiveness is written as Equation (14):

(14) $$D=\frac{MI_{F}}{\overset{m}{\underset{j=1}{\text{∑}}}MI(C_{j},F_{j})}$$

The contrast value indicates the *reliability* property directly. In addition, the power of reliability can also be estimated by the record times that the feature has been successfully re-observed. Thus the quantitative characterization of reliability can be measured according to Equation (15):

(15) $$R=W_{H}H_{\text{norm}}+(1-W_{H})\frac{m}{M}$$

where *H_norm_* denotes the normalized Hessian value (SURF key-point), and *W_H_* is the weight factor.

The feature strength value *V_str_*, given in Equation (16), is obtained by adding the three quantitative characterizations:

(16) $$V_{\text{str}}=W_{M}MI_{\text{norm}}+(1-W_{m}-W_{R})D_{\text{norm}}+W_{R}R$$

where *MI_norm_* is the normalized mutual information, *D_norm_* is the normalized reliability, *W_M_* and *W_R_* are the mutual information and the reliability weight factors, respectively.

### 3.2. Feature Updating

As for the long-range navigation in changing environments, the feature updating is a matter of cardinal significance. The approaches close to our work are presented in [9,10]. They are both based on the multi-store model of human memory [28], which is divided into three basic stores: *sensory memory* (SM), *short-term memory* (STM) and *long-term memory* (LTM). The sensory memory stores all information perceived by the senses, and elementary selection measures are taken to determine what information will be transported to the STM. Through a process of rehearsal, the information in STM can be transported to LTM to be retained for longer periods of time. The approach presented in [9] is mainly used to keep feature sets updating, and the feature upgrade or degradation is based on feature “hit” or “miss” rather than quantitative characterization. An adapted memory model is adopted to update the features in [10], where the main purpose is robot localization.

In this paper, the feature updating is implemented by adopting the concepts mentioned above in a simple way. During the process of SURF feature extraction, original key-points are denoted as SM, and those with low contrast and large principal curvature will be rejected. Then the final features are classified as STM or LTM according to their strength values. For the purpose of finding the most suitable threshold *T_str_* to distinguish STM from LTM, an experiment described specifically in Section 5.1 is conducted to test the homing performance. When the feature strength values are re-computed, the feature sets will be updated. For STM, they have four times to be transported to LTM, on the premise that their strength values are greater than the threshold *T_str_*, otherwise, they will be deleted. Besides, the LTM will degrade to STM if the strength values are less than the threshold *T_str_*.

## 4. Overview of the Proposed Feature Optimization Procedure

**Figure 2.**
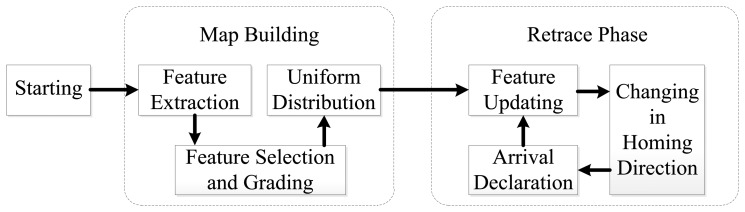
Outline of feature optimization procedure.

The outline of the feature optimization procedure of long-range visual homing is shown in Figure 2. The route consisting of nodes is determined when the robot enters the environments for the first time. When the robot retraces the environments, it follows the route using the strategies in [2–6]. During the retracement process, the ALV homing algorithm directs the robot towards the target node. In addition, the ALV homing direction is computed with the assistance from an external compass. The two-stage procedure is detailed as follows:

(1) Map building. The robot is manually driven in the environments and panoramic images are captured at particular places with approximately 2 m between each other. Each node in the route is a collection of optimal features, which are extracted from the panoramic image. In the first step, each panoramic image is divided into regular sector rings as shown in Figure 1, and features are extracted by standard SURF algorithm. Then the strength value is calculated for each feature, and all features are classified into STM and LTM according to their strength values.
  For all the LTM features, the method proposed in Section 2 is used to improve the feature distribution, regardless of the STM features. If the LTM feature number in one sector ring is less than the number required, the STM features with higher strength values compensate for the shortage of feature number. The uniformly distributed LTM features are used to compute the ALV vectors.
(2) Retrace phase. When the robot follows the succession of nodes by ALV homing method, the feature updating approach will be adopted.

During the navigation between two nodes, panoramic images are continuously captured and matched with the image taken at the target node before the robot arrives at the node. The strength values of features stored at the target node are re-evaluated during this process. Therefore, the feature set can be updated. It should be noticed that feature matching aims at re-evaluating the strength values rather than calculating homing direction, since there is no need of feature matching in the ALV method.

The homing direction will be changed in two cases. One case is that, if the LTM features used to compute current direction of migration change by *T_num_* or higher, the ALV vector at current location will be computed to obtain new homing direction. The other case is that, if the LTM features stored at the target node is updated, its ALV vector will be re-computed and the robot will move on in a new direction.

With regard to route guidance using linked local visual homing, there are two issues of crucial importance that have to be noticed: the determination of a new node and the arrival detection at a node [3,7,8]. As part of autonomous navigation, the major objective of this work is to maintain feature optimization, thus, the determinations on at which point a new node is set and whether the robot is sufficiently close to the target node are not the key points. In this paper, the locations of nodes are manually selected at a distance of approximately 2 m. Besides, the arrival detection of nodes is straight. During navigation between nodes, features extracted at current position are continuously matched with features stored at the target node. Arrival is declared once the maximum value of the bearing angle differences of matching features falls below threshold *T_θ_*.

## 5. Experiments

### 5.1. Robot Platform and Parameter Settings for Experiments

**Figure 3.**
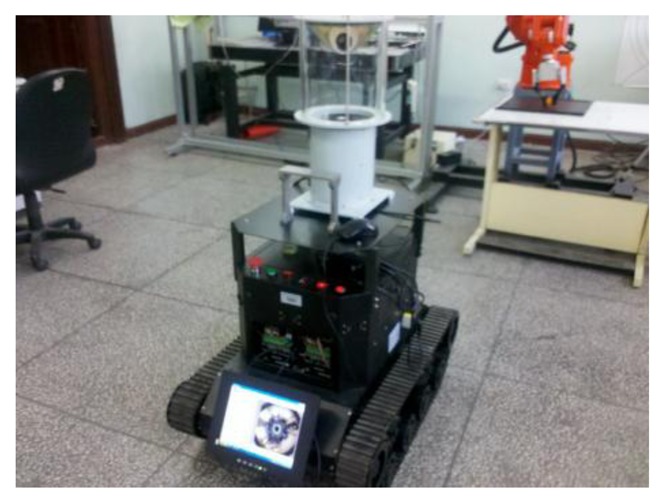
The mobile robotic platform.

Our experiments were performed with a crawler mobile robot (see Figure 3). A panoramic vision system, composed of a mirror with a diameter of 100 mm and a camera with a resolution of 800 × 800, was mounted on top of the robot. A 2 GHz Pentium (R) processor was dedicated to vision processing and robot control. In addition, the robot was equipped with a digital compass to acquire the global orientation.

Table 1 shows the parameter settings. Similar to the standard SURF algorithm, the scale space consists of three octaves and four scale layers (*O* = 3 and *S* = 4). Because the number of features decreases gradually with the number of octaves, the number of sector rings *n_os_* should taper off. More specifically, the sector ring number in each scale layer of the first octave is 100 (5 × 5 × 4), and the others are 64 (4 × 4 × 4) and 36 (3 × 3 × 4). The weight factor *W_D_* in Equation (6) must be determined by an appropriate compromise between the spatial dispersion and feature strength value. The optimal value of *W_D_* was determined by a local homing experiment, where we used 60 random pairs of panoramic images captured in the room and corridor. When the homing error was calculated, the Hessian value was substituted for feature strength value in Equation (6). The value assigned to *W_D_* was changed from 0 to 1 in 0.1 step. By seeking the minimum average angular error of homing, 0.3 was determined as the optimal value. The same experiment was conducted to determine the optimal value of *W_H_*, and the only difference was that the reliability value was substituted for feature strength value in Equation (6). Finally *W_H_* was set to 0.6 through the experimental analysis. Similar to the above two experiments, another experiment was performed to figure out the most suitable *W_M_* and *W_R_* values. The feature strength value measured according to Equation (16) was used. The minimum average angular error of homing was obtained when *W_M_* value was 0.5 and *W_R_* value was 0.3.

**Figure 4.**
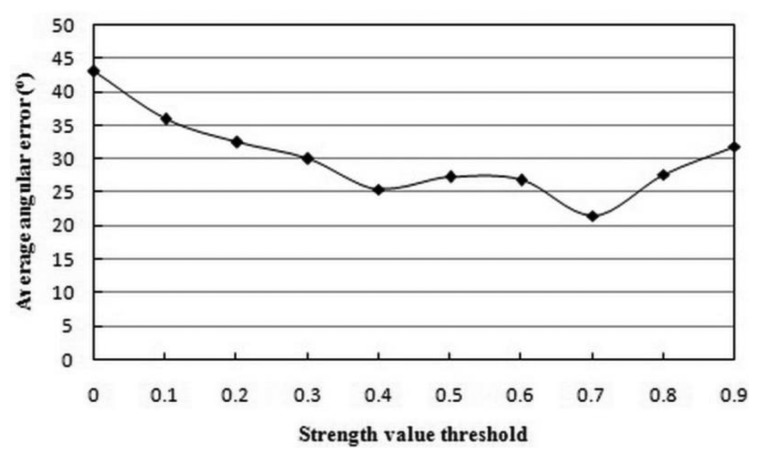
Average angular error versus strength value threshold.

We conducted another experiment to find the optimal threshold to distinguish LTM features from STM features. The threshold *T_str_* must balance feature quantity and quality. A low threshold will result in redundant features, and the features with low relative importance will degrade the homing performance. On the contrary, a high threshold will prune too many features, giving rise to the lack of representativeness of environmental appearance. The STM features will be less accessible to turn into LTM ones. The experimental results are shown in Figure 4. A well-established compromise in the mutual contradiction mentioned earlier was reached when the threshold *T_str_* was set to 0.7. In addition, the thresholds *T_num_* for changing homing direction and *T_θ_* for arrival detection were empirically set to 10% and 10°, respectively.

### 5.2. Evaluation Tests of the Uniformly Distributed Features

**Figure 5.**
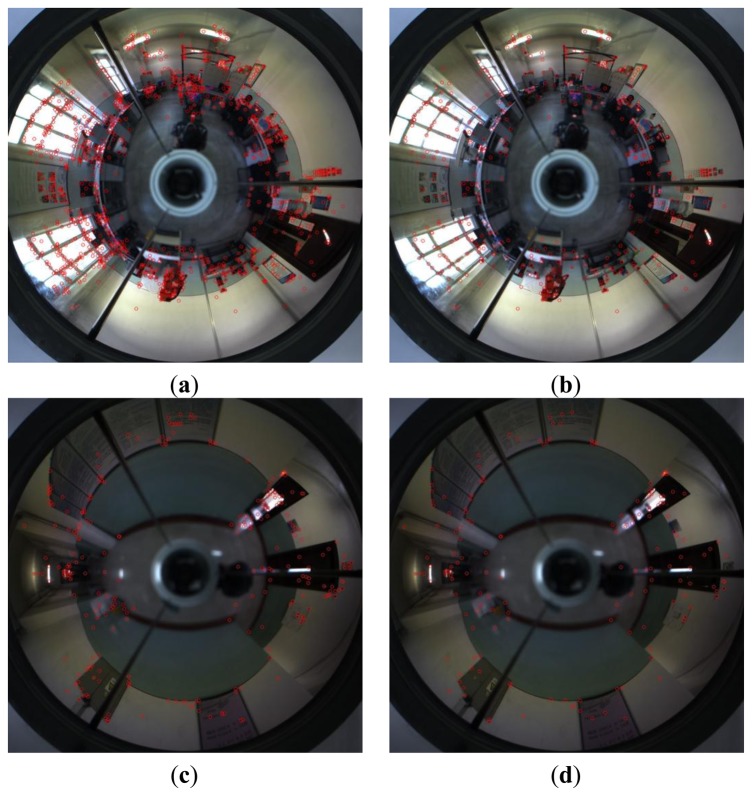
The distribution of features. The red circles denote feature positions. (**a**,**c**) show the non-uniform distribution of features extracted by the standard SURF; (**b**,**d**) show the uniform distribution of features extracted by the proposed approach.

To evaluate the improvement on feature distribution using the proposed approach, an experiment was conducted in the room with a 10 m × 5 m floor space and the corridor with a 16 m × 3 m floor space. Figure 5a,c show the distribution of features extracted by the standard SURF algorithm. They manifest that the feature distribution is non-uniform, which means that the homing performance will be degraded. Figure 5b,d show the feature extraction results by the proposed modified version of SURF algorithm. The comparison shows the feasibility of the proposed approach in the improvement on feature distribution. However, this is just visual inspection. The improvement on the distribution quality should be judged in terms of visual homing. The chosen measure of quantitative characterization of distribution, similar to the one proposed in [29], is defined as:

(17) $$Q_{\text{dist}}=\frac{1}{O\times S}\overset{O}{\underset{o=1}{\text{∑}}}\overset{S}{\underset{s=1}{\text{∑}}}\left(-\overset{n_{os}}{\underset{i=1}{\text{∑}}}p_{i}\log_{2}\;p_{i}\right)$$

where *p_i_* is the ratio of matching features in the *i*th sector ring over all the ones in the scale layer (*s*) of the octave (*o*). If the correctly matching features were uniformly distributed, a maximum distribution quality would be achieved (5.94 for our experiments).

**Figure 6.**
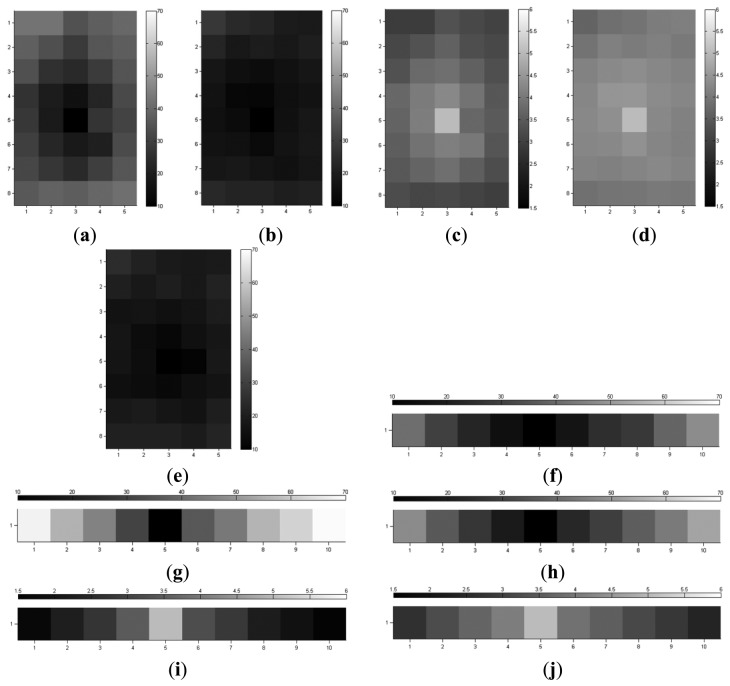
The homing error and distribution quality. The axes denote the positions where the homing errors are computed. (**a**,**b**) show the homing error in the room using features extracted by the standard SURF and the proposed approach, respectively; (**c**,**d**) show the distribution quality corresponding to the results in (**a**,**b**); (**e**,**f**) show the homing error in the room and the corridor, respectively using the method in [4]; (**g**,**h**) show the homing error in the corridor using features extracted by the standard SURF and the proposed approach, respectively; (**i**,**j**) show the distribution quality corresponding to (**g**,**h**).

Figure 6 shows the homing error and the corresponding feature distribution quality. Homing error is the absolute angular error between correct homing vector and the computed homing vector. In addition, the homing method presented in [4] was taken for comparison, as shown in Figure 6e,f. The proposed approach performed as well as the method in [4], with slight performance differences. The promising results indicate that it is feasible to improve the homing accuracy of ALV by employing the proposed approach, which can decrease the impact of anisotropic feature distribution.

### 5.3. Long-Range Homing Experiments

**Figure 7.**
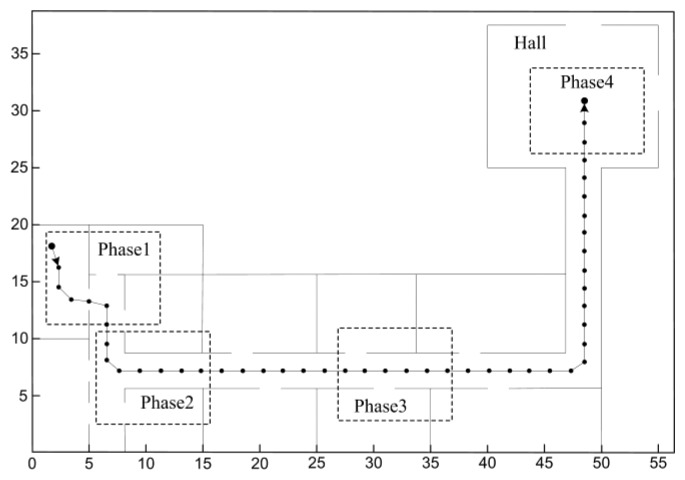
The experimental environments. Phase 1 denotes the visual homing process in the room and the junction between the room and corridor; phase 2 corresponds to the process in the junction between two corridors; phase 3 denotes the process in the corridor; phase 4 corresponds to the process in the hall.

In this section, long-range homing experiments were conducted to demonstrate the feasibility of the feature optimization approach, and the standard SURF algorithm was taken for comparison. Figure 7 shows the environment representation marked with four typical regions, where no special arrangements and modifications were made to facilitate the execution of visual homing. The robot was located upon the starting node, and sequentially passed the intermediate nodes until it arrived at the home node eventually. Notice that the initial orientations of the robot were roughly the same in the following experiments.

The experiments were classified into several groups according to the degree of change of the environmental appearance. The environments were manually changed in various manners, including modifying illumination conditions by control of curtains and lamps, rearranging object locations, adding new objects, removing existing objects and covering objects with cardboard. It should be noticed that temporary occlusions caused by people always existed. Here, five types of experiments had been conducted: in the first one, under good illumination conditions, the environmental layout remained the same as that in the first tour without modification; the second one was conducted under worse illumination conditions, compared with the first one; in the third and fourth experiments, environment changes were identical, and the illumination conditions were the same as that in the first and second, respectively; the fifth one was conducted in randomly changing environments. By means of the proposed feature optimization approach, the features stored in nodes updated five times via the five types of experiments mentioned above.

**Figure 8.**
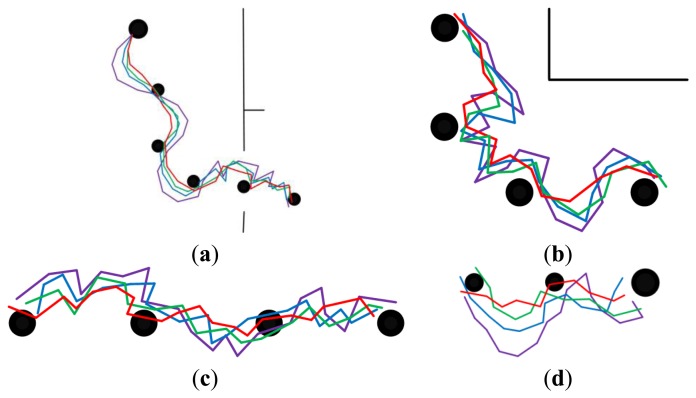
The homing trajectories in non-modified environments with fairly good illumination. (**a**–**d**) show the results obtained in phase 1, 2, 3 and 4, respectively. Red lines correspond to the proposal. Violet lines correspond to SURF. Blue lines correspond to the proposal without selection and updating. Green lines correspond to the proposal without updating. In each figure, the robot moves from left to right, the black disks with the same size are intermediate nodes, and the bigger one is starting node or home node.

**Figure 9.**
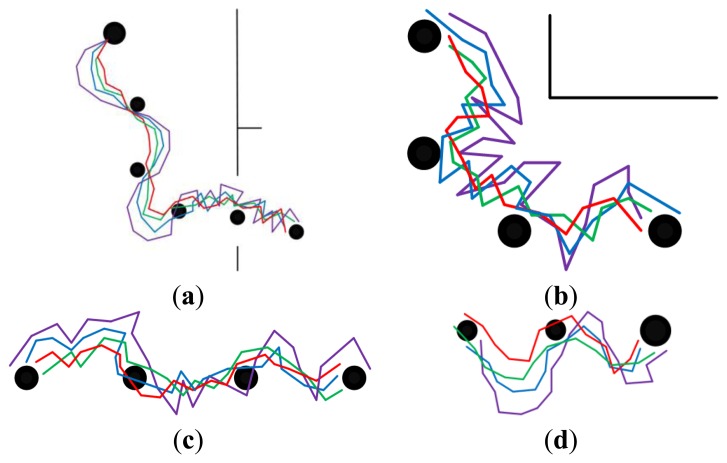
The homing trajectories in non-modified environments with relatively poor illumination. (**a**–**d**) show the results obtained in phase 1, 2, 3 and 4, respectively. Red lines correspond to the proposal. Violet lines correspond to SURF. Blue lines correspond to the proposal without selection and updating. Green lines correspond to the proposal without updating. In each figure, the robot moves from left to right, the black disks with the same size are intermediate nodes, and the bigger one is starting node or home node.

**Figure 10.**
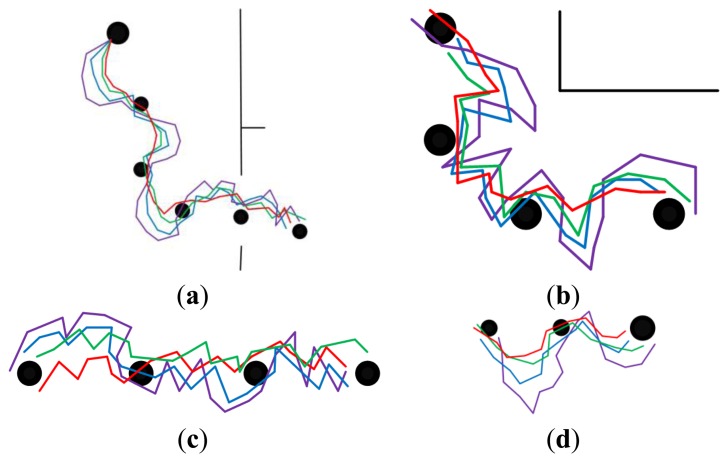
The homing trajectories in modified environments with fairly good illumination. (**a**–**d**) show the results obtained in phase 1, 2, 3 and 4, respectively. Red lines correspond to the proposal. Violet lines correspond to SURF. Blue lines correspond to the proposal without selection and updating. Green lines correspond to the proposal without updating. In each figure, the robot moves from left to right, the black disks with the same size are intermediate nodes, and the bigger one is starting node or home node.

**Figure 11.**
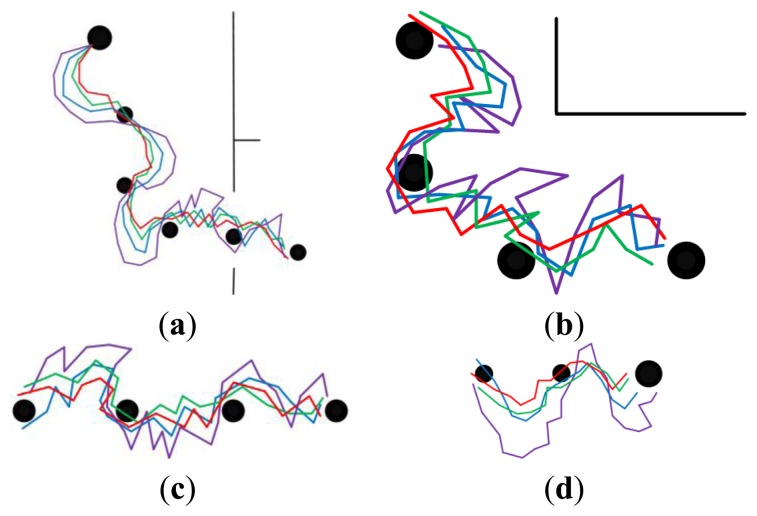
The homing trajectories in modified environments with relatively poor illumination. (**a**–**d**) show the results obtained in phase 1, 2, 3 and 4, respectively. Red lines correspond to the proposal. Violet lines correspond to SURF. Blue lines correspond to the proposal without selection and updating. Green lines correspond to the proposal without updating. In each figure, the robot moves from left to right, the black disks with the same size are intermediate nodes, and the bigger one is starting node or home node.

**Figure 12.**
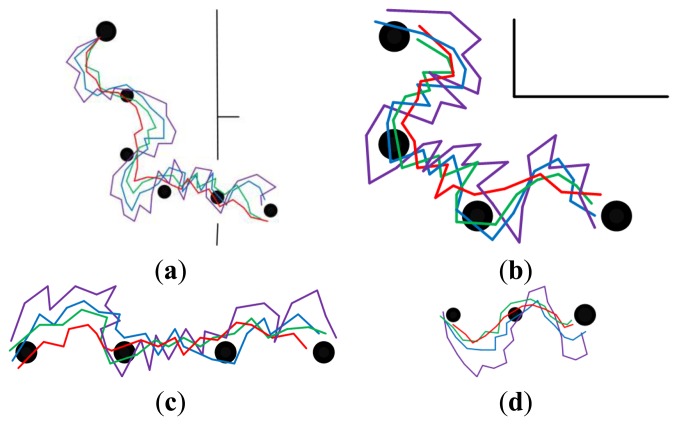
The homing trajectories in randomly changing environments. (**a**–**d**) show the results obtained in phase 1, 2, 3 and 4, respectively. Red lines correspond to the proposal. Violet lines correspond to SURF. Blue lines correspond to the proposal without selection and updating. Green lines correspond to the proposal without updating. In each figure, the robot moves from left to right, the black disks with the same size are intermediate nodes, and the bigger one is starting node or home node.

In order to demonstrate the effectiveness of each part of the feature optimization procedure, our approach was compared with three other approaches. The experimental results are presented in Figures 8,9, 10,11–12. The results of the proposed approach and the standard SURF are shown by red and violet, respectively. The blue indicates the results obtained by our approach without the procedure of feature selection and updating. And the results shown by green are obtained by our approach without the procedure of feature updating. The decrease of curvature and the improvement on smoothness can be chosen as criteria to the effectiveness of the proposed approach on homing accuracy.

#### 5.3.1. Long-Range Homing with Different Illumination Conditions in Non-Modified Environments

Figures 8 and 9 show the homing trajectories under different illumination conditions in non-modified environments. Figure 8 corresponds to the results obtained at around noon with fairly good illumination, and Figure 9 corresponds to the results obtained in the afternoon with relatively poor illumination. It can be observed that the experimental results obtained under fairly good illumination condition are better than those obtained under relatively poor illumination conditions. Observing the results of the four approaches, our approach outperforms the other three ones. The performance of the standard SURF is the worst, which may be caused by its poor control of the feature distribution and quality. The performance differences between the two experimental results are relatively bigger when using the standard SURF, compared with the other three approaches. This can be attributed to the utilization of feature selection and updating, which partially decrease the influence of large changes in illumination and people. Moreover, it is noteworthy that the homing performance in the corridor is improved apparently owing to the uniformly distributed features, compared to that in the room and hall. Although the performance is improved in the corridor, the homing error is bigger compared with that in the room and hall. Besides, a problem has been arisen that there exists significant degradation of homing performance in the junctions of two corridors, between the room and corridor, since the environmental appearance information between them is obviously different.

#### 5.3.2. Long-Range Homing with Different Illumination Conditions in Modified Environments

The experimental results under different illumination conditions in modified environments are provided in Figures 10 and 11. Figure 10 indicates the results obtained at around noon with fairly good illumination, and Figure 11 is obtained at around afternoon with relatively poor illumination. The modifications made in the environments will result in decreasing of matching feature number, thus the ability of coping with the large changes in environments can be tested. Analyzing the homing trajectories, our approach performs well despite the changes in environmental layout. Though the homing error caused by anisotropic feature distribution decreases when the robot approaches the target node, the performance differences demonstrate that the feature optimization procedure does take effect. In a word, our approach maintains a better appearance representation of the changing environments.

#### 5.3.3. Long-Range Homing in Randomly Changing Environments

Figure 12 shows the homing trajectories under randomly changing environmental conditions. The illumination conditions were changed randomly by controlling the curtains and lamps, and the object positions were in disorder, posing a challenge to visual homing. Comparing the experimental results with those in Sections 5.3.1 and 5.3.2, it is clear that the performance differences increase as the feature set updating times goes up. It is worth to note that the approach using standard SURF is incapable of dealing with these situations. From the experimental results, the effect of the feature optimization in our approach, which is the result of these performance differences, is evident. Given these challenging conditions, our approach performs well and demonstrates that it is of importance to get a suitable appearance representation of the environments.

### 5.4. Analysis of Experimental Results

From the perspective of route following, the homing trajectories in this paper are not the straightest, compared with those in [30–32]. Considering the simple navigation framework, the homing error tends to decrease as the robot is guided closer to the target node. Besides, the method of selecting node locations may result in the lack of features in common between consecutive nodes. Therefore, the homing direction will be changed frequently due to the strategy of changing mobile direction. The changing environments, the extreme unevenness of feature distribution in the corridor, and the transition from one environment to another with obviously different appearance pose severe challenges to visual homing.

To quantify the performance, the mean values and standard deviations of the mobile direction angles during the long-range homing are listed in Table 2, where the smoother trajectory and better performance is indicated by lower mean value and standard deviation. From the homing trajectories and mobile direction angles, the feature optimization method improves the homing accuracy. However, it is hard to know how well the proposed method performs due to the error introduced by the digital compass. During the first tour when the route was determined, the images were captured when the robot was parallel to one side of the oblong floor tile. Therefore, the directions of translation between images stored at neighboring nodes are known. The homing errors between neighboring nodes can be computed using the feature sets obtained through the above five experiments, without impact of the digital compass. The results in Table 3 indicate that each part of the optimization procedure has a positive effect on the decrease of homing error.

Despite the homing performance improvement, the equal-distance assumption is difficult to meet completely. This is obvious in the corridor and can be explained in the way that features are projected on the panoramic images [13]. In addition, the mechanisms of arrival detection and new node determination in this paper do not perform very well. The robot does wrongly declare the node arrival in a number of cases. The set distance of 2 m between consecutive nodes does not work in every environment and results in lack of autonomy.

## 6. Conclusions

This paper proposes an approach to feature optimization, especially for long-range feature-based homing in changing environments. The task-dependent optimization procedure consists of feature distribution, selection and updating. The method proposed in this paper shows good homing performance, including improvement on feature distribution quality, homing accuracy and environmental adaptability. Therefore, it is feasible and suitable for long-range navigation in changing environments.

The selection of key images is significant for representing a route with fewer nodes. In addition to that, the arrival detection is closely related to homing accuracy. However, the locations of nodes are determined manually and arrival detection mechanism does not work well in this paper. As crucial parts of autonomous navigation, more efforts need to be spent on solving these two issues. Other possible improvements lie in computing the most suitable strength value threshold with an adaptive algorithm to distinguish STM features from LTM features, as well as exploring the possibility of substituting visual compass for inaccurate digital compass.

## Figures and Tables

**Table 1.** Parameters in the proposed approach.

**Parameters**	**Value**	**Parameters**	**Value**	**Parameters**	**Value**
*O*	3	*n_4s_*	16	*T_str_*	0.7
*S*	4	*W_D_*	0.3	*T_num_*	10%
*n_1s_*	100	*W_H_*	0.6	*T_θ_*	10°
*n_2s_*	64	*W_M_*	0.6		
*n_3s_*	36	*W_R_*	0.3		

**Table 2.** Mobile direction angles. Experiments 1–5 correspond to the results shown in Figures 8,9,10,11–12, respectively. *E* (°) denotes the mean value of mobile direction angle, and *σ* (°) denotes the standard deviation of mobile direction angle. Compared with the standard SURF (violet trajectory), *P_E_* and *P_σ_* denote the percentages by which mean value and standard deviation have been lowered through the five experiments, respectively.

**Trajectories**	**Experiment 1**		**Experiment 2**		**Experiment 3**		**Experiment 4**		**Experiment 5**		***P****_E_*	***P****_σ_*

	***E***	***σ***	***E***	***σ***	***E***	***σ***	***E***	***σ***	***E***	***σ***		
_Violet_	41.4	10.8	50.3	12.5	51.6	12.8	57.9	13.3	59.5	13.9	0	0
_Blue_	29.3	6.6	34.7	7.8	33.9	7.5	37.2	8.3	40.5	8.7	32.4%	38.6%
_Green_	23.8	5.0	27.6	5.9	28.3	6.2	32.2	6.9	34.5	7.3	43.8%	50.7%
_Red_	18.1	3.9	20.5	4.5	21.6	4.6	23.1	4.8	25.3	5.2	58.2%	63.7%

**Table 3.** Average homing errors. Feature sets 1–5 correspond to the features stored at the nodes and obtained during the above five types of experiments. Compared with the standard SURF (violet trajectory), *P* denotes the percentage by which the homing error has been lowered through the five feature sets.

**Approach**	**Feature Set 1**	**Feature Set 2**	**Feature Set 3**	**Feature Set 4**	**Feature Set 5**	***P***
Violet	45.1	56.3	53.7	59.8	63.4	0
Blue	31.2	35.4	36.1	38.9	41.5	34.0%
Green	22.6	27.1	29.5	33.9	35.4	46.9%
Red	17.6	21.1	23.8	25.4	26.5	59.0%
